# Supplementary material for: In vivo imaging using surface enhanced spatially offset raman spectroscopy (SESORS): balancing sampling frequency to improve overall image acquisition
Source: Npj Imaging. Author manuscript; Available in PMC 2024 Jun 27. (PMC11210722; doi:10.1038/s44303-024-00011-9)
Supplement: Supplementary Information [file NIHMS1995991-supplement-Supplementary_Information.pdf]

Supporting Information

In vivo Imaging using Surface Enhanced Spatially Offset Raman Spectroscopy (SESORS): Balancing Sampling Frequency to Improve Overall Image Acquisition

Fay Nicolson[1,2]*, Bohdan Andreiuk[2,3], Eunah Lee[4], Bridget O'Donnell[4,5], Andrew Whitley[4], Nicole Riepl[6], Deborah L. Burkhart[1], Amy Cameron[2], Andrea Protti[2], Scott Rudder[7], Jiang Yang[8], Samuel Mabbott[9,10], Kevin M. Haigis[1]

1. Department of Cancer Biology, Dana-Farber Cancer Institute and Harvard Medical School, Boston, MA 02215, USA
2. Department of Imaging, Dana-Farber Cancer Institute and Harvard Medical School, Boston, MA 02215, USA
3. Cancer Immunology and Virology, Dana-Farber Cancer Institute and Harvard Medical School, Boston, MA 02215, USA
4. HORIBA Instruments Incorporated, Piscataway, NJ 08854, USA
5. Honeywell International Inc., Fort Washington, PA 19034, USA
6. College of Science, Northeastern University, 360 Huntington Avenue, Boston, MA 02115
7. Innovative Photonic Solutions, Monmouth Junction, NJ, 08852, USA
8. State Key Laboratory of Oncology in South China, Collaborative Innovation Center for Cancer Medicine, Sun Yat-sen University Cancer Center, Guangzhou, China
9. Department of Biomedical Engineering, Texas A&M University, Emerging Technologies Building, College Station, TX, 77840, USA
10. Center for Remote Health Technologies & Systems, Texas A&M Engineering Experiment Station, 600 Discovery Drive, College Station, TX, 77840, USA

* Corresponding Author:

Fay Nicolson, Department of Cancer Biology, Dana-Farber Cancer Institute and Harvard Medical School, Boston, MA 02215, USA,

Email: fay_nicolson@dfci.harvard.edu

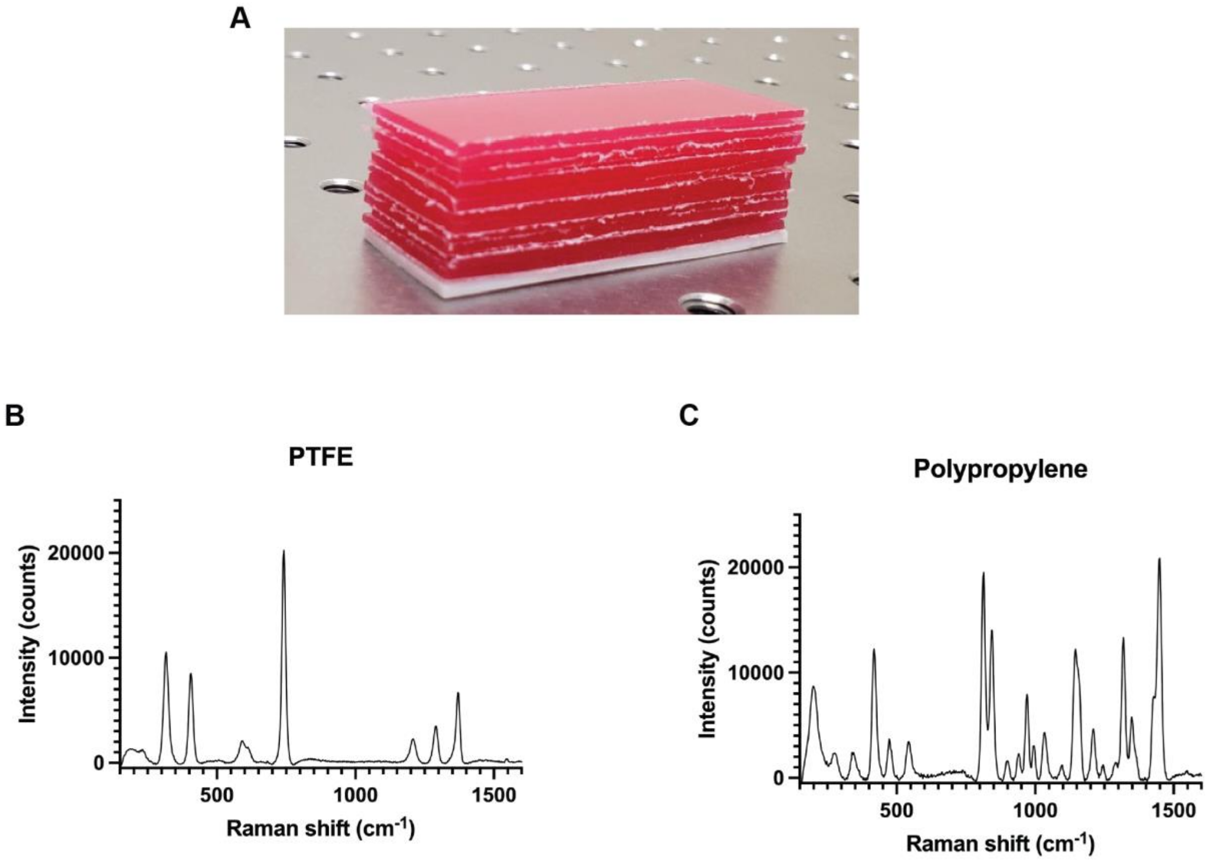

Figure S1: Calibration standards and their corresponding Raman spectrum. (A) Sheets of pink polypropylene (PP) were placed on top of white polytetrafluoroethylene (PTFE) to create calibration standards. (B) Raman spectrum of PTFE. (C) Raman spectra of PP. Spectra were acquired using a 785 nm laser, 1s integration time.

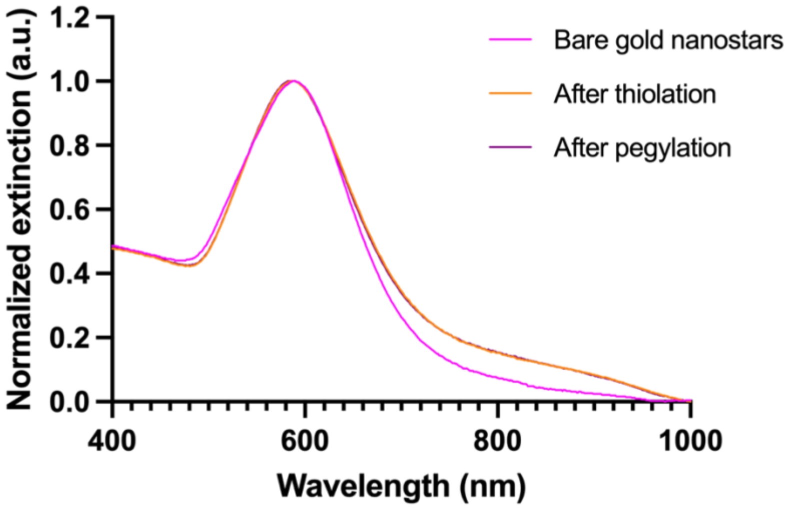

Figure S2: Extinction spectroscopy of SERRS CAs after thiolation and after PEGylation. Spectra were characterized using a UV-2600 UV-VIS spectrophotometer (Shimadzu).

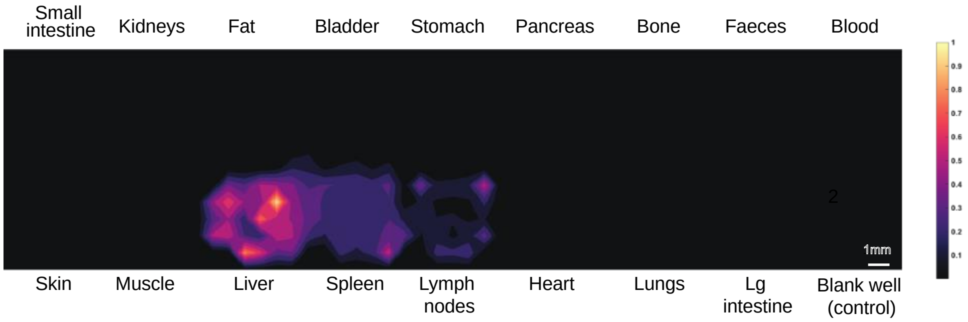

Figure S3: Biodistribution of SERRS CAs in tissues. GL261 tumor-bearing mice (n=3) were injected with 100 µL of 2 nM of SERRS nanostars, and tissues of interest harvested and homogenized. The tissues were then analyzed by Raman imaging (25% laser power, 785 nm laser, 1 s acquisition time, 5× objective) to determine the relative accumulation in different tissues. Images are representative of n = 3 mice, 1 organ per well. Measurements were acquired using a LabRAM HR Evolution (HORIBA Scientific).

Table S1: SSIM values obtained for comparison images and plotted as a heat map. Values were obtained using SESORRS in vivo images acquired using a par-sampling frequency approach and an over sampling frequency of 2, 5 and 10.

Image 1 (sampling frequency approach)	Image 2 (sampling frequency approach)	SSIM value
Par-sampling	Over-sampling x2	0.678
Par-sampling	Over-sampling x5	0.575
Par-sampling	Over-sampling x10	0.490
Over-sampling x2	Over-sampling x5	0.738
Over-sampling x2	Over-sampling x10	0.622
Over-sampling x5	Over-sampling x10	0.767

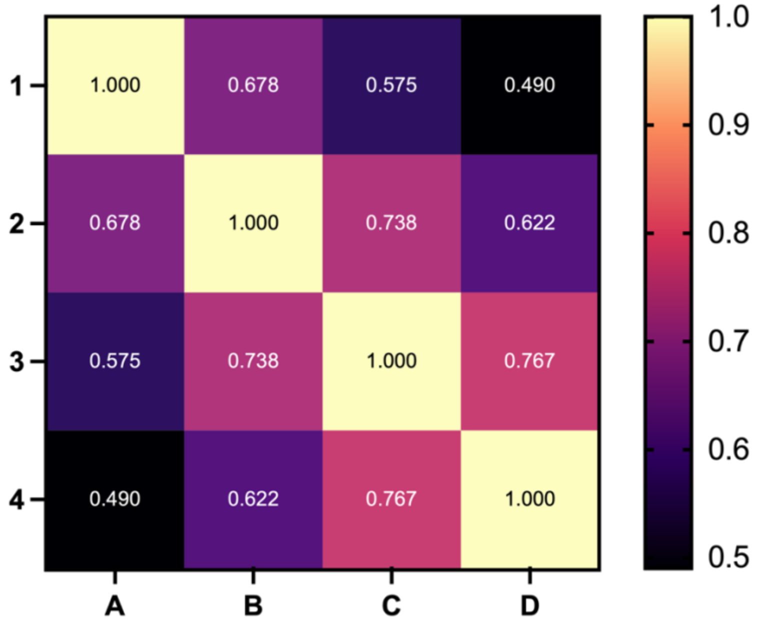

Figure S4: Heat map showing the differences in SSIM. SSIM considers changes in the texture, luminance, and contrast rather than just pixel-by-pixel differences. SSIM ranges from -1 to 1, where 1 indicates that the two images being compared are identical, and -1 indicates that there is no structural similarity whatsoever. The heat map was plotted using the SSIM values in Table S1.
